# The Wheat Starchy Endosperm Protein Gradient as a Function of Cultivar and N-fertilization Is Reflected in Mill Stream Protein Content and Composition

**DOI:** 10.3390/foods12234192

**Published:** 2023-11-21

**Authors:** Wisse Hermans, Justine Busschaert, Yamina De Bondt, Niels A. Langenaeken, Christophe M. Courtin

**Affiliations:** Laboratory of Food Chemistry and Biochemistry & Leuven Food Science and Nutrition Research Centre (LFoRCe), KU Leuven, Kasteelpark Arenberg 20, B-3001 Heverlee, Belgium; wisse.hermans@kuleuven.be (W.H.); yamina.debondt@kuleuven.be (Y.D.B.); niels.langenaeken@kuleuven.be (N.A.L.)

**Keywords:** milling, protein distribution, gluten, break fractions, sub-aleurone, N-fertilization, wheat varieties, flour quality

## Abstract

Within the wheat starchy endosperm, the protein content increases biexponentially from the inner to outer endosperm. Here, we studied how this protein gradient is reflected in mill fractions using three cultivars (Claire, Apache, and Akteur) grown without and with N-fertilization (300 kg N ha^−1^). The increasing protein content in successive break fractions was shown to reflect the protein gradient within the starchy endosperm. The increasing protein content in successive reduction fractions was primarily due to more aleurone contamination and protein-rich material being harder to reduce in particle size. The miller’s bran fractions had the highest protein content because of their high sub-aleurone and aleurone content. Additionally, the break fractions were used to deepen our understanding of the protein composition gradient. The gradient in relative gluten content, increasing from inner to outer endosperm, was more pronounced without N-fertilization than with and reached levels up to 87.3%. Regarding the gluten composition gradient, no consistent trends were observed over cultivars when N-fertilization was applied. This could, at least partly, explain why there is no consensus on the gluten composition gradient in the literature. This study aids millers in managing fluctuations in the functionality of specific flour streams, producing specialized flours, and coping with lower-quality wheat.

## 1. Introduction

Wheat (*Triticum aestivum* L.) accounts for 19% of global protein intake, making it the number one protein source worldwide [1]. A wheat kernel comprises four main parts: the botanical bran (pericarp, testa, and nucellar epidermis) (6–8% *w*/*w*), aleurone layer (6–7% *w*/*w*), starchy endosperm (80–84% *w*/*w*) and embryo (3% *w*/*w*) [2,3]. The starchy endosperm is spatially heterogeneous in architecture and composition [4,5]. Small, protein-rich sub-aleurone cells can be found at its periphery [5,6,7]. The prismatic endosperm cells are located beneath the sub-aleurone cells, extend towards the center of the cheeks, and measure 128–200 μm × 40–60 μm × 40–60 µm [5]. The central endosperm cells lie in the center of the cheeks, are round or polygonal, low in protein, and have dimensions of 72–144 μm × 69–120 μm [5]. The compositional heterogeneity within the starchy endosperm includes a gradient in protein content and protein composition from the center of the cheeks towards the boundary with the aleurone layer [4,6,7,8,9]. The gradient in protein content can be described as an ascending biexponential curve with protein contents as high as 32% beneath the aleurone layer due to the presence of protein-rich sub-aleurone cells [7]. The extent of this gradient highly depends on the cultivar and the level of nitrogen fertilization (N-fertilization) [7].

Millers aim to separate the starchy endosperm from the aleurone layer and botanical bran and grind this into white flour using roller milling, a dry fractionation process. Using multiple sets of corrugated break rolls, smooth reduction rolls, and plansifters or sieves, more than 50 white flour fractions [10] and 10 bran-rich side streams are produced in an industrial mill. Fluctuations in the functionality of specific flour streams, stemming from differences in the starchy endosperm protein gradient, may introduce variability in the miller’s end products. At the same time, these fluctuations also create opportunities for the development of customized flour formulations. Insight into how the protein gradient in the endosperm is reflected in mill streams is, therefore, of utmost importance.

In the scientific literature, laboratory roller mills are often used. They operate on a similar principle as industrial mills but vary in terms of the number of flour- and bran-rich fractions they yield, as well as their efficiency (flour yield). Pairs of corrugated break rolls open the kernels and scrape off the resulting bran particles to remove the adhering starchy endosperm [11]. The pairs of smooth reduction rolls reduce the starchy endosperm particles to fine flour [11]. It was reported that the last break (BFs) and reduction fractions (RFs) are higher in protein content compared to the other flour fractions [12,13]. This is often ascribed to aleurone and botanical bran contamination [12,14]. However, Tosi et al. [14] hypothesized that the lower protein content of the first BF and RF could be attributed not only to lower levels of bran contamination but also to the fact that these could originate from the inner endosperm, while the later BFs and RFs would be derived from the outer endosperm. Simmons and Sutton [15] and Wang et al. [16] observed that the increase in protein content from the first to last BF was more outspoken than throughout the RFs. To the best of our knowledge, there is currently no literature available on the protein content of mill fractions derived from different cultivars grown at different levels of N-fertilization, and, therefore, also displaying a high variability in gradient in protein content within the starchy endosperm.

In addition to the protein content, the protein composition plays a crucial role in determining the functionality of flour streams [17]. Two important aspects concerning protein composition are the relative gluten content (the proportion of proteins being gluten proteins) and the gluten composition (the relative proportion of gluten protein types) [4].

Sequential pearling of kernels is not very well suited for studying the gradient in relative gluten content due to its limited spatial specificity [4,18]. The non-spherical shape of wheat kernels and the crease cause outer endosperm fractions to be contaminated with aleurone material, which does not contain gluten [19]. Recently, Hermans et al. [9] used laser microdissection to separate sub-aleurone tissue from inner endosperm tissue and showed with proteomics that the sub-aleurone has a significantly higher relative gluten content than the inner endosperm. Although this study effectively detected significant differences in relative gluten content, it could not provide accurate mass-based relative gluten contents [9]. However, it did give a very strong indication that the gradient in relative gluten content increases from the inner to outer endosperm. Unlike the gradient in relative gluten content, sequential pearling of kernels can be used to investigate the gradient in gluten composition. However, the results reported by Tosi et al. [6] and He et al. [8] were inconclusive. For example, for various wheat cultivars and varying levels of N-fertilization, the proportion of high molecular weight glutenin subunits (HMW-GS) (expressed relative to total gluten content) was either observed to be higher, lower, or not significantly different in the sub-aleurone-enriched fraction compared to the inner endosperm-enriched fractions [6,8]. In both pearling studies, the presence of aleurone contamination could also interfere with the SDS-PAGE and SE-HPLC analysis performed in these studies, as the molecular weight of aleurone proteins ranges from <10 kDa to near 100 kDa [20]. Hermans et al. [9] did not detect a significant difference in HMW-GS protein proportion between the sub-aleurone and inner endosperm. Hence, there is no consensus on the gradient in gluten composition, while there are strong indications that the gradient in relative gluten content increases from the inner endosperm toward the sub-aleurone, although the magnitude of this increase remains unclear.

Limited research has been conducted regarding the protein composition of mill fractions. However, there are indications that the relative gluten content changes throughout both the BFs and RFs [15,16]. Regarding gluten composition, no differences were noted across the BFs and RFs [15,16]. However, Wang et al. [16] did document an increase in the proportion of gliadins across the RFs.

Because of the industrial relevance and the identified gaps in the existing literature, the main goal of this study was to investigate how the protein gradient within the starchy endosperm, affected by cultivar and N-fertilization, is translated to the protein content and composition of mill fractions. To investigate this, three wheat cultivars, grown with and without N-fertilization, of which the gradient in protein content was analyzed by Hermans et al. [7], were milled. The resulting mill fractions were subsequently investigated in detail. The findings of this study are relevant for both fundamental research and the milling industry. Given the trend toward less N-fertilization (imposed by governments) for environmental reasons, investigating the effect of N-fertilization on the protein content and composition of mill streams is extremely relevant. Due to reduced N-fertilization, industrial millers are required to produce functional ingredients from lower-quality raw materials. This capability, in turn, could accelerate the transition to reduced N-fertilization. 

## 2. Materials and Methods

### 2.1. Materials

Sodium dihydrogen phosphate dihydrate, sodium phosphate dibasic dodecahydrate, TRIS, hydrochloric acid, 1-propanol, dithiothreitol (DTT), and acetonitrile were procured from Sigma-Aldrich (Bornem, Belgium), trifluoroacetic acid (TFA) and absolute ethanol (99.8% *v*/*v*) from Thermo Fisher Scientific (Aalst, Belgium), and sodium chloride and urea from VWR International (Oud-Heverlee, Belgium). All chemicals were at least of analytical grade. 

### 2.2. Sample Set

Wheat (*Triticum aestivum* L., Poaceae) of three cultivars (Claire (Cl), Apache (Ap), Akteur (Akt)) was cultivated at the experimental site Brakumdreef (Lubbeek, Belgium). Each cultivar was cultivated without (0 kg N ha^−1^) or with N-fertilization (a total amount of 300 kg N ha^−1^). The N, which was applied in solid form as calcium ammonium nitrate (27% N), was equally distributed over tillering and the beginning of stem elongation. All information regarding the cultivation of the wheat is described in Hermans et al. [7]. The wheat was harvested in August 2017 and subsequently cleaned to remove impurities such as stones and residual husks.

These three wheat cultivars were chosen because they differ in protein characteristics and, therefore, suitability for bread making. Claire is a class C cultivar, which is considered unsuitable for bread making. Apache is a class B cultivar suitable for bread making when mixed with higher classes. Akteur is classified in class E, the most suitable for bread making. Moreover, for the six wheat samples used in this study, the gradient in protein content within the starchy endosperm has been determined by Hermans et al. [7]. Finally, the difference in protein composition between the sub-aleurone and inner endosperm of Akteur, grown at 300 kg N ha^−1^, was studied by Hermans et al. [9] using laser microdissection and nanoLC-MS/MS. 

### 2.3. Wheat Milling

Wheat kernels were milled using a Bühler MLU-202 laboratory mill (Bühler group, Uzwil, Switzerland) [21]. Kernels of the cultivars Akteur and Apache were tempered to a moisture content of 16.5%, whereas kernels of the cultivar Claire were tempered to a moisture content of 16.0%. A schematic representation of the Bühler MLU-202 laboratory mill is given in Figure S1 (Supplementary Materials). The mill produces six flour fractions, a miller’s bran fraction, and a shorts fraction. The first pair of break rolls exert a shearing action to open the kernels and separate inner endosperm portions from the remainder of the kernel rather than crushing them. Subsequently, the second and third pair of break rolls scrape off the bran particles to remove the adhering starchy endosperm [11]. After each pair of break rolls, there is a set of sieves, eventually leading to three break flour fractions (BFs). BF1 only passed the first pair of break rolls, while BF3 passed all three pairs of break rolls. Miller’s bran particles are sieved out (>530 μm) after the third set of break rolls. The gaps between the first, second, and third pair of break rolls were set at 0.35 mm, 0.18 mm, and 0.07 mm, respectively. Particles that are too large to end up in the BFs (>150 µm) but do not end up in the miller’s bran fraction are sent to three pairs of smooth reduction rolls, which, after sieving, lead to three reduction flour fractions (RFs). The shorts are sieved out after the third pair of reduction rolls. The gaps between the first, second, and third pair of reduction rolls were set at 0.08 mm, 0.06 mm, and 0.04 mm, respectively. All milling trials were performed in duplicate. After the determination of the yields, the duplicate fractions were mixed.

### 2.4. Chemical Analysis

The protein content of the kernels and mill fractions was determined according to an adaptation of the AOAC Official Method 990-03 (AOAC International, 1995) to an automated micro-DUMAS protein analysis system (Vario Max Cube, Elementar, Hanau, Germany). The number 5.70 was chosen as the N-to-protein conversion factor for all samples (kernels, flour, and miller’s bran). That choice disregards small differences that could derive from the milling fractions exhibiting variations in protein composition due to differences in their starchy endo-sperm protein-to-aleurone and botanical bran protein ratios, but also due to differences in cultivar, level of N-fertilization, and the part of the starchy endosperm from which they originate. For the kernels, shorts fractions, and miller’s bran fractions, the N determination was preceded by a milling step with a CryoMill (RETSCH, Haan, Germany; grinding mode: dry at room temperature) to obtain a fine, homogeneous sample because only 1 mg of sample is needed for the micro-DUMAS system. The ash content was determined following an adaptation of the AACC International method 08-01.01 (AACC International, 1999) [22]. The moisture content was determined according to the AACC International method 44-15-02 (AACC International, 1999). The ash and moisture content measurements were performed in triplicate. The protein content was analyzed in duplicate at least. 

### 2.5. Light Microscopy

Mill streams were embedded in Historesin, as described by Dornez et al. [23]. Cross sections of 1 μm thickness were prepared with a rotary microtome HM355 (MicromLabergerate GmbH, Walldorf, Germany). These sections were transferred to microscopy slides (Thermo Scientific Cel-line, SSG Braunschweig, Germany), and proteins were stained with 0.1% (*w*/*v*) Naphthol Blue Black in 7% (*v*/*v*) acetic acid in water for 2 min. Finally, bright-field images were captured at 100× magnification with a Nikon Eclipse 80i microscope (Nikon Inc., New York, NY, USA). 

### 2.6. Modified Osborne Fractionation and Analytical RP-HPLC

Wheat proteins were separated into albumins and globulins, gliadins, and glutenins using a modified Osborne fractionation. Afterward, RP-HPLC was performed to determine the relative amounts of these fractions and the relative amounts of different gluten types (ω-, α- and γ-gliadins, D-low-molecular-weight glutenin subunits (LMW-GS) (sulfur-poor), B/C-LMW-GS (sulfur-rich) and HMW-GS) [18,24,25]. Albumins and globulins were separated, starting from an amount of sample corresponding to 100 mg protein, by conducting two sequential extractions with a 3.0 mL salt solution (sodium phosphate buffer in water (0.05 M; pH 7.6) with 0.4 M NaCl) and a third extraction with 3.0 mL water. Each extraction (10 min) was followed by a centrifugation step (10 min, 4500× *g*). The three supernatants were combined, diluted to 10 mL with the extraction buffer, and filtered (0.45 mm). Gliadins were extracted from the remaining pellet by performing three sequential extractions with ethanol/water (60/40, *v/v*) according to the same procedure. Subsequently, glutenins were extracted similarly using 50% aqueous 1-propanol containing Tris-HCl (0.05 M, pH 7.5), urea (2 M), and 1% DTT at 60 °C for 20 min under a nitrogen atmosphere. All extracts were loaded (2 µL) on an Aeris C18 column (3.6 µm, 150 × 2.1 mm, Phenomenex, Torrance, CA, USA) using an HPLC system (Shimadzu, Kyoto, Japan) consisting of an LC-20AT pump, a DGU-20A5 degasser, a SIL-20ACHT autosampler, a CTO-20AC column oven, and an SPD-10AVP UV/VIS detector. The oven was set at 60 °C and UV/VIS detector at 214 nm. The mobile phases MilliQ (Milli-Q Plus, Merck Millipore, Darmstadt, Germany) water (A) and acetonitrile containing 0.1% (*v*/*v*) TFA (B) were used at a flow rate of 0.3 mL/min, following a linear gradient from 24% B to 90% B in 70 min. The proportions of albumins and globulins, gliadins, and glutenins were calculated from their respective area compared to the total area of all protein fractions. The proportion of gluten types, ω-, α- and γ-gliadins, HMW-GS, and LMW- GS were deduced from the corresponding areas relative to the total area of the gliadin or glutenin fraction. Examples of the chromatograms obtained by modified Osborne fractionation and RP-HPLC are given in Figure S2 (Supplementary Materials). Samples were analyzed in triplicate.

### 2.7. Statistical Analysis

A Tukey’s honest significant difference (HSD) test was applied to detect statistically significant differences between means. Principal component analysis (PCA) was performed to transform a high-dimensional dataset into a new coordinate system. Regression models were fitted using the standard least squares method. The effects (main, two-way interaction, and quadratic effects) included in the models were determined using backward elimination and F-tests. The significance level was set at 5%. The coefficients of determination (R² and R²_adjusted_ (R²_adj_)) of the predicted models were calculated. The statistical analysis was performed using JMP Pro 17.0.0 software (SAS Institute, Cary, NC, USA).

## 3. Results and Discussion

### 3.1. Effect of Kernel Protein Content on the Wheat Milling Yields

The cultivar and level of N-fertilization are the major factors determining the protein characteristics of wheat. Hence, we chose to work with three cultivars from different baking classes, each grown at 0 and 300 kg N ha^−1^. The high variability in protein characteristics is already reflected in the kernel protein content, which varies from 7.2% to 14.2% (Figure S3 (Supplementary Materials)). The cultivar Claire has the lowest protein content, while Akteur has the highest. This was expected as Claire was chosen because of its low suitability and Akteur for its high suitability for bread making. Note that the differences between cultivars increase if N-fertilization is applied, confirming their different N-use efficiency.

**Figure 1:**
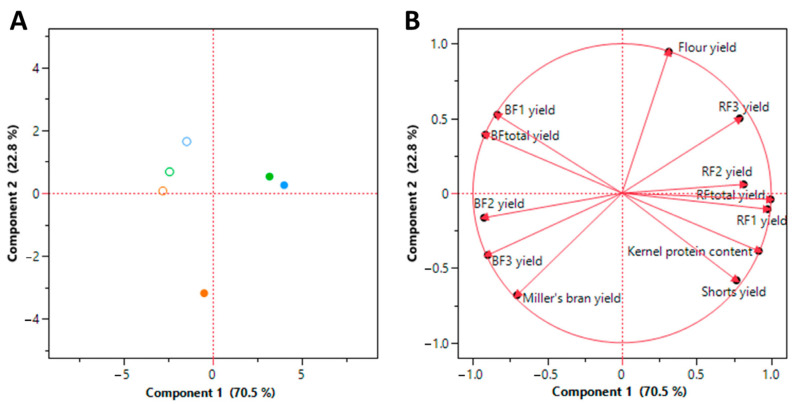
Principal component analysis (**A**) score and (**B**) loading plot of the kernel protein content and milling yields. BF: break fraction, RF: reduction fraction. Principle component 1 explains 70.5% of the variability and principle component 2 22.8%. Orange: Claire, Green: Apache, Blue: Akteur. ○: 0 kg N ha^−1^, ●: 300 kg N ha^−1^.

The wheat kernels of the cultivars Claire, Apache, and Akteur, each cultivated at 0 and 300 kg N ha^−1^, were milled using a laboratory roller mill. The mill produces six flour fractions, a miller’s bran fraction, and a shorts fraction. The six flour fractions include three BFs and three RFs. The yields of these eight fractions are displayed in Table 1. The flour yields vary from 62.8% to 69.5%. As expected, these are significantly lower than the 72% to 80% yield achieved by industrial millers [4,11]. However, the kernel protein content appears to determine the milling behavior of the kernels. To establish the relationships between the kernel protein content and milling yields, a PCA was performed (Figure 1). In a PCA loading plot, positive relations between variables are indicated by arrows in the same direction, and inverse relations by opposing arrows (Figure 1B). The total flour and miller’s bran yield (varying from 26.8% to 32.3%) are not significantly correlated with the kernel protein content. The yield of the three break fractions combined (BF_total_), varying from 20.3% to 34.6%, is inversely correlated with the kernel protein content (R^2^ = 0.96). This means that 96% of the variation in the BF_total_ yield can be explained by the variation in kernel protein content, and more specifically, the yield of BF_total_ decreases by 2.1% for each 1% increase in kernel protein content. The variation in BF_total_ yield is mainly caused by the variation in BF_1_ yield (Figure 1B). The yields of the reduction fractions combined (RF_total_) (varying from 33.5% to 48.6%) and the shorts fractions (varying from 1.8% to 4.5%) are positively correlated with the kernel protein content, having an R^2^ of 0.84 and 0.75, respectively. Hence, higher protein contents seem to make the starchy endosperm harder. If the starchy endosperm, separated from the bran by the corrugated break rolls, is harder to reduce in particle size, there will be more large particles. As these cannot pass the sieves leading to the BFs, the yield of the BFs decreases, and more material is transferred to the reduction rolls, increasing the yields of the RFs. Additionally, the yields of the shorts increase because the material transferred to the reduction rolls is harder to reduce in particle size. Hourston et al. [26] and Kent [27] previously addressed the relationship between starchy endosperm hardness and protein content.

### 3.2. How Is the Protein Gradient within the Starchy Endosperm Reflected in the Mill Streams?

**Figure 2:**
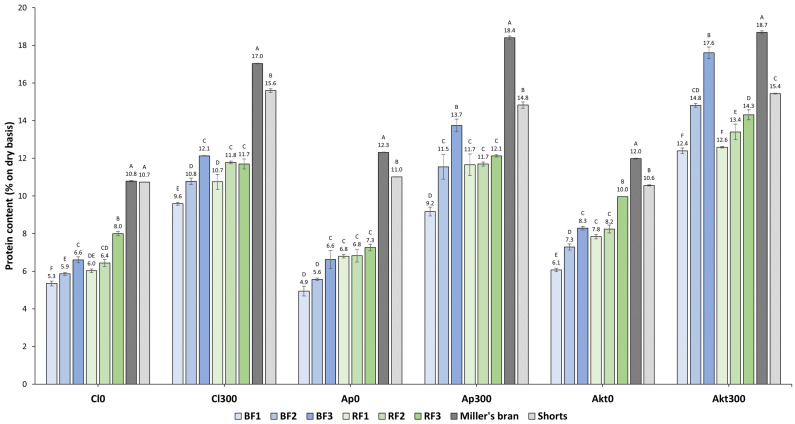
Protein contents of the mill streams of the cultivars Claire (Cl), Apache (Ap), and Akteur (Akt) grown with and without N-fertilization (300 kg N ha^−1^). BF: break fraction, RF: reduction fraction. Labeling with different letters points out significant differences (Tukey’s HSD test, *p* ≤ 0.05) between mill fractions of one cultivar grown at one N-fertilization level.

The protein contents of the eight mill streams of the three cultivars, grown without and with N-fertilization (300 kg N ha^−1^), are displayed in Figure 2. If no N-fertilization is applied, an increase in protein content is observed from BF1 to BF3, on the one hand, and RF1 to RF3, on the other hand. Both increases are of comparable magnitude. For example, for Cl0, the protein content rises 1.3% (or a relative increase of 23.5%) in the BFs, while 2.0% (or a relative increase of 32.8%) in the RFs. This is very interesting as in the absence of N-fertilization, Claire, Apache, and Akteur exhibit very similar and gentle gradients in protein content [7]. From the inner endosperm to the sub-aleurone, the protein content of Cl0 increased from 6.3 ± 1.0% to 14.0 ± 3.7%, of Ap0 from 6.7 ± 0.7% to 18.2 ± 3.8% and of Akt0 from 5.9 ± 1.0% to 19.1 ± 4.5% [7].

**Figure 3:**
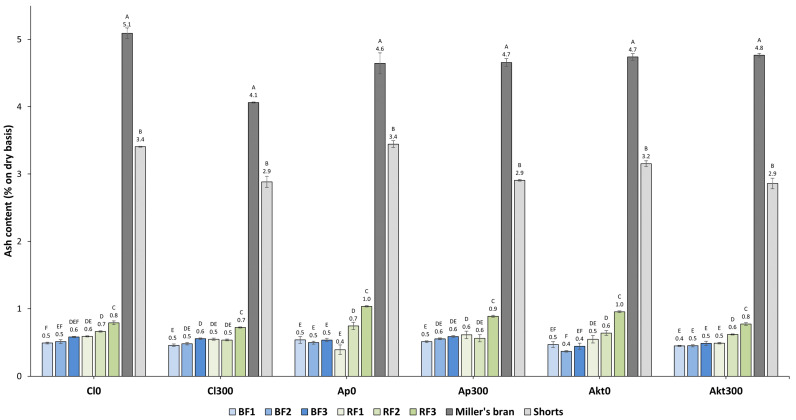
Ash contents of the mill streams of the cultivars Claire (Cl), Apache (Ap), and Akteur (Akt) grown with and without N-fertilization (300 kg N ha^−1^). BF: break fraction, RF: reduction fraction. Labeling with different letters points out significant differences (Tukey’s HSD test, *p* ≤ 0.05) between mill fractions of one cultivar grown at one N-fertilization level.

If N-fertilization (300 kg N ha^−1^) is applied, the gradients in protein content within the starchy endosperm are notably steeper and also differ between the cultivars, with Akteur having the steepest gradient [7]. From the inner endosperm to the sub-aleurone, the protein content of Akt300 increased from 9.8 ± 1.6% to 31.7 ± 3.3%, compared to 8.7 ± 0.4% to 24.4 ± 2.3% for Cl300 [7]. Analyzing the mill streams when N-fertilization is applied, similar to when no N-fertilization is applied, an increase in protein content is also observed across both the BFs and RFs. However, in this case, the increase in the protein content in the BFs is substantially higher than in the RFs. For example, for Akt300, the protein content in the BFs rises by 5.2% (or a relative increase of 42.0%), whereas in the RFs, it increases by only 1.7% (or a relative increase of 13.6%). In addition, we observed that, with N-fertilization, the increases in protein content in the RFs are lower or comparable to the increases in protein content across the RFs without N-fertilization. Hence, these increases in the reduction fractions are independent of the gradient in protein content and are hypothesized to be solely the result of protein-rich material being harder to reduce in particle size. The rise in protein content in the BFs is significantly higher for the 300 kg N ha^−1^ samples compared to the samples without N-fertilization, with the rise being the lowest for Cl300 and highest for Akt300. This is highly comparable with their gradients in protein content [7]. Given the mechanism of action of the break rolls, this is a strong indication that the gradient in protein content is reflected in the BFs. However, the increasing protein content in the BFs could also be caused by increasing levels of contamination with aleurone, which contains approximately 30% protein [28]. Given the aleurone’s high ash 315 content of approximately 12%, ash content can be used as a marker for aleurone contamination [29,30]. The ash contents of the mill fractions are displayed in Figure 3. As expected, the miller’s bran and shorts fractions, which contain elevated aleurone levels, exhibit significantly greater ash content than the flour fractions. Across the RFs, the ash content increases significantly for each of the six samples. This indicates that the rises in protein content in the RFs do not solely stem from the protein-rich material being harder to reduce in particle size but also from increased levels of aleurone contamination. Except for Cl300, none of the samples show a significant increase in ash content across the BFs. The values of 0.4 to 0.6% are also representative of commercial flours. This demonstrates that the increase in protein content in the BFs is not caused by increasing levels of aleurone contamination and, therefore, confirms our hypothesis that the gradient in protein content is reflected in the BFs.

**Figure 4:**
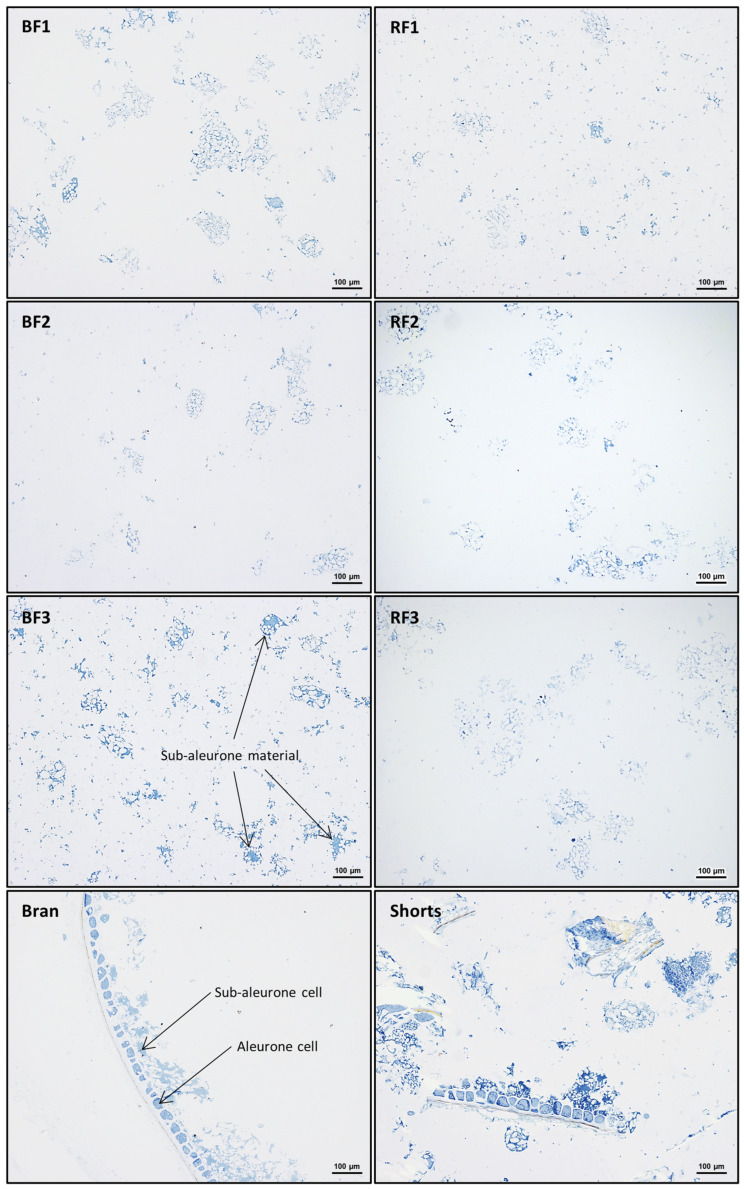
Microscopy images of the mill streams of Akteur, grown at 300 kg/ha, in which proteins are stained with Naphthol Blue Black. BF: break fraction, RF: reduction fraction.

This is also demonstrated by microscopy. In Figure 4, microscopy images of the eight mill fractions of Akt300 are shown. Proteins are stained blue with Naphthol Blue Black. In the BFs and RFs, one can observe the typical starchy endosperm material, high in starch granules, embedded in a protein matrix. Compared to the other flour fractions, BF3 also contains a notable amount of large protein aggregates. These are characteristic of the subaleurone cells, lying at the periphery of the starchy endosperm. However, the significant amount of sub-aleurone material still present in the miller’s bran fraction shows the incomplete recovery of the starchy endosperm in the flour fractions. This is also revealed by analyzing the obtained flour yields, ranging from 62.8% to 69.5% (Table 1), because the starchy endosperm constitutes 80–84% of a wheat grain [2,3]. This means that only a part of the periphery of the starchy endosperm is recovered in the flour fractions, mainly in BF3, and the remaining part remains attached to the bran. This, together with the high protein content of the aleurone, also explains why the miller’s bran fraction has the highest protein content of all the mill fractions (Figure 2). Between samples, the miller’s bran protein content follows a similar trend as the kernel protein content. For the shorts, no clear cultivar-dependent effects can be distinguished.

To conclude, there are strong indications suggesting that BF1 is enriched in the inner endosperm, BF2 in the more distant starchy endosperm, and BF3 in the peripheral starchy endosperm. However, it is not possible to precisely delineate the specific starchy endosperm region to which each BF corresponds, and there is likely an overlap between the starchy endosperm regions present in the three BFs. Furthermore, a part of the sub-aleurone remains adhered to the miller’s bran fraction, preventing it from being included in BF3. 

This reflection of the gradient in protein content in the BFs provides opportunities. The BFs obtained in this study could be used to gain insight into the gradient in protein composition and its dependence on cultivar and level of N-fertilization. However, there are some limitations. The incomplete recovery of the starchy endosperm, coupled with the vague and intertwined boundaries of the starchy endosperm regions corresponding to each BF, leads to an underestimation of the gradient in protein content within the starchy endosperm. This is shown by comparing the gradient in protein content of Akt300, measured in detail by Hermans et al., to the rise in protein content in the BFs, which, respectively, increase from 9.8 to 31.7% [7] and 12.4 to 17.6% (Figure 3). Consequently, this will similarly affect the estimation of the gradient in protein composition.

### 3.3. Analysis of the Protein Composition Gradient within the Starchy Endosperm Using the Break Fractions

The protein composition of the BFs of the cultivars Claire, Apache, and Akteur, grown with and without N-fertilization (300 kg N ha^−1^), is shown in Table 2. Over the different BFs, cultivars, and N-fertilization levels, the relative gluten content varies from 76.61% to 87.29%. Statistical modeling revealed that the factors ‘break fraction’, ‘cultivar’, and ‘N-fertilization’, as well as their interactions, significantly affect the relative gluten content (R² _adj_ = 0.95). A visual representation of the estimated model is given in Figure S4A (Supplementary Materials). Here, the parameter estimates are presented in descending order of absolute value, from highest to lowest. The relative gluten content is the lowest for the cultivar Claire and the highest for Akteur, and for each cultivar, N-fertilization increases the relative gluten content. Akteur, having the highest relative gluten content, reaffirms its high suitability for baking purposes. Furthermore, the relative gluten content significantly increases from BF1 to BF3, and hence, from the inner to outer endosperm, across all cultivars at each level of N-fertilization, except for Cl300 (Table 2). This confirms the results of Hermans et al. [9] and also of Simmons and Sutton [15] and Wang et al. [16]. For Akt300, the relative gluten content even increased to 87.29% in BF3 (Table 2), but, taking into account the underestimation (described in Section 3.2), this is expected to be substantially higher in the sub-aleurone cells.

The increase in relative gluten content throughout the BFs, and hence, from the inner to outer endosperm, appears to be independent of the cultivar but is affected by the level of N-fertilization. The increase in relative gluten content throughout the BFs is of a higher magnitude without N-fertilization compared to with N-fertilization. This was according to expectations because a basic machinery, i.e., a basic set of albumins and globulins, is hypothesized to be present throughout the entire starchy endosperm [9]. In other words, once the basic set of albumins and globulins is built up, the excess of N is predominantly accumulated as gluten proteins. For example, for Akt0, BF1 and BF3 have a protein content of 6.07% and 8.29%, respectively. In BF1, the absolute gluten content (percentage of gluten proteins on total dry weight) is 4.76%, and the non-gluten protein content is 1.30%, while for BF3, those are 6.81% and 1.48%, respectively. Hence, the difference in absolute gluten content between BF1 and BF3 is 2.05%, whereas it is only 0.17% in non-gluten protein content. This shows that the 2.22% extra accumulated proteins in BF3 compared to BF1 are almost all gluten proteins, which confirms the hypothesis that once the basic set of albumins and globulins is built up, the excess of N is predominantly accumulated as gluten proteins. Increasing the level of N-fertilization will, therefore, mainly lead to additional accumulation of gluten proteins in both the inner and outer endosperm. This will level out the gradient in relative gluten content as in the inner endosperm, with the lower relative gluten content, there is more potential for an increase compared to the outer endosperm with the higher initial relative gluten content. Assuming that increasing the level of N-fertilization from 0 to 300 kg N ha^−1^ only leads to extra accumulation of proteins, we can calculate that 89.5%, 89.8%, and 91.0% of the extra accumulated proteins are gluten in BF1, BF2, and BF3 of Akteur, respectively. Considering that this study provides underestimations of the magnitudes of the gradients in protein composition, we can hypothesize that, for the subaleurone cells, more than 91.0% of the proteins additionally accumulated due to N-fertilization are gluten proteins. Taking this further, one could expect that the additional protein accumulation in the sub-aleurone, going from a high level of N-fertilization, e.g., 150 kg N/ha, to an even higher level, e.g., 300 kg N/ha, is almost purely due to the additional accumulation of gluten proteins for the cultivar Akteur. Important to note here is that not only relatively speaking, more gluten proteins are being accumulated in the outer endosperm due to higher levels of N-fertilization, but also in absolute numbers. All this confirms the hypothesis that the sub-aleurone cells act as storage facilities for “excess” N.

Regarding gluten composition, Table 2 shows that the three cultivars vary in their proportion of gluten types. Claire exhibits a relatively high amount of γ-gliadins, whereas Akteur is characterized by a high proportion of HMW-GS. Consequently, the LMW-GS/HMW-GS ratio is the lowest for the cultivar Akteur. The gliadin-over-glutenin (GLIA/GLU) ratio, on the other hand, decreases from Claire, over Apache, to Akteur (Table 2, Figure S4B (Supplementary Materials)). For both bread-making indicators, the LMW-GS/HMW-GS and GLIA/GLU ratio, low values are desired, and this once again highlights the high baking quality of the cultivar Akteur. Note that for GLIA/GLU and LMW-GS/HMW-GS, ‘cultivar’ is the most determining factor, while for the relative gluten content, ‘break fraction’ is (Figure S4B (Supplementary Materials)). In general, the effect of N-fertilization seems to be dependent on the cultivar. However, more N-fertilization consistently results in a higher proportion of ω-gliadin and a decrease in the LMW-GS/HMW-GS ratio (Figure S4C (Supplementary Materials)).

From BF1 over BF2 to BF3, the trends in gluten composition appear to depend on the cultivar–N-fertilization combination. However, some general trends can be observed. In the absence of N-fertilization, there is a trend of increasing ω- and γ-gliadin and decreasing B/C-LMW-GS and HMW-GS proportions from BF1 to BF3, and therefore, from the inner to outer endosperm (Table 2). As a result, the GLIA/GLU ratio significantly increased from BF1 to BF3 for Cl0 and Ap0. This can also be observed in Figure S4B (Supplementary Materials), displaying a visual representation of the model estimating the GLIA/GLU ratio in function of the cultivar, level of N-fertilization, and BF (R² _adj_ = 0.89). The decrease in the proportion of HMW-GS from the inner to outer endosperm was also reported by He et al. [8] at low N levels (100 kg N ha^−1^) and Tosi et al. [6]. The increase in the proportion of ω-gliadin was only observed at 350 kg N ha^−1^ by He et al. [8]. He et al. [8] did not individually quantify the proportions of α-gliadin, γ-gliadin, and LMW-GS. At 300 kg N ha^−1^, no consistent trends were observed. The gradient in proportion of certain gluten types decreased, increased, or remained constant depending on the cultivar. He et al. [8] conducted similar observations for the proportion of LMW-GS and gliadins combined and HMW-GS at 350 kg N ha^−1^ of N-fertilization. Therefore, it seems that different cultivars exhibit similar gradients in gluten composition in the absence of N-fertilization, while there is more variation at high levels of N-fertilization. This may partially explain the (seemingly) contradictory literature on the gradient in gluten composition. 

Finally, in the current study, the differences in gluten composition between the inner endosperm and sub-aleurone, as observed in Akt300 by Hermans et al. [9] through the utilization of cryosectioning and nanoLC-MS/MS, did not manifest between BF1 (enriched in inner endosperm) and BF3 (enriched in peripheral endosperm). Hermans et al. [9] demonstrated that gluten of the sub-aleurone contains a higher proportion of ω-gliadins but a lower proportion of LMW-GS and γ-gliadins compared to the inner endosperm. The inability to detect this here may be attributed to two factors: firstly, analyzing the BFs provides an underestimation of the actual gradients (described in Section 3.2), and secondly, this study analyzes gluten proteins differently. The current study classifies gluten proteins according to extractability and hydrophobicity into gluten types like γ-gliadins, HMW-GS, etc., while Hermans et al. [9] use protein sequences for classification into gluten types. These methods do not lead to perfect one-to-one matches [31].

### 3.4. Relevance of Sub-Aleurone in the Milling Industry

Knowledge of how the protein gradient within the starchy endosperm is reflected in the mill streams can be very relevant for the milling industry and lead to new opportunities. From previous research, we know that for Cl0, the protein content increased from about 6.3% in the inner endosperm to 14.0% in the sub-aleurone. For Akt300, this rise was from about 9.8% to 31.7% [7]. Integrating this with the current study on the composition of mill streams can lead to new insights. Making the conservative assumption that the relative gluten content of BF1 corresponds to that of the inner endosperm and that of BF3 to that of the sub-aleurone, the relative gluten content increases from 76.6% to 81.2% across the starchy endosperm of Cl0 and from 84.5% to 87.3% for Akt300 (Table 2). Combining the results on the gradient in protein content and protein composition, the absolute gluten content (relative to total dry weight) increases from 4.8% in the inner endosperm to 11.4% in the sub-aleurone for Cl0 and 8.3% to 27.7% for Akt300. While this is an underestimation, it demonstrates the enormous potential of the sub-aleurone as a gluten source. Given the dependence of both the gradient in protein content and protein composition on the wheat cultivar and level of N-fertilization, even higher values are possible for other cultivar-N-fertilization combinations. However, knowing that in this study, BF3 of Akt300 contains 17.6% protein, of which 87.3% gluten proteins, we can hypothesize that at least 1 of the more than 50 flour streams of an industrial mill contains more than 20% protein of which more than 90% is gluten, due to the presence of sub-aleurone material. This fraction(s) could be used in specialized flours. 

Due to the trend towards less N-fertilization (imposed by governments), the production of functional ingredients from low-quality raw materials is an immense challenge. However, this study shows that we can lower the level of N-fertilization while maintaining a high protein content and favorable protein composition just by selecting mill streams. For example, when lowering the level of N-fertilization, and hence, also the quality of the wheat, the high-protein flour streams could be collected to produce flour for bread making, whereas the flour streams with a lower protein content are well-suited for the production of, e.g., cookies, cakes, and coatings. This can be one of the components contributing to the transition to reduced N-fertilization, along with increasing wheat’s nitrogen use efficiency (NUE) through breeding, soil health management, implementing precision agriculture practices, etc. 

Industrial mills never achieve full recovery of the starchy endosperm. This means that at least some sub-aleurone material, which is the part of the kernel that is most enriched in gluten, remains adhered to the miller’s bran. Despite this, most miller’s bran (about 90%) ends up in animal feed [32]. This creates big opportunities. Nowadays, there is no selection procedure prior to using miller’s bran for whole-meal bread making. Hence, one opportunity lies in selecting miller’s bran based on its sub-aleurone protein properties before its incorporation into whole-meal products like bread. We hypothesize that, for example, Akt300 bran will perform better in whole-meal bread making than Cl0 bran. Additionally, further research is required to explore the separation of sub-aleurone material adhered to the bran from the bran itself. This is in line with the goal of promoting sustainable N management, where both increasing wheat’s NUE and incorporating protein from side streams into human food are crucial. Both are essential for alleviating the environmental impact of N-fertilization. Finally, while this study provides insight into the effect of cultivar (genetics) and N-fertilization (element of environment) on the gradient in protein composition, more research is needed to map the impact of environmental factors.

## 4. Conclusions

This study is the first to systematically investigate how the protein gradient within the starchy endosperm, influenced by cultivar and N-fertilization, translates into the protein content and composition of mill fractions. Both within the BFs and RFs, an increase in protein content was observed. The increase in protein content within the RFs could be attributed to increased levels of aleurone contamination and protein-rich material being harder to reduce in particle size. The rise in protein content within the BFs was shown to be a reflection of the gradient in protein content within the starchy endosperm. This implies that BF1 is enriched in the inner endosperm, BF2 in the more distant starchy endosperm, and BF3 in the peripheral starchy endosperm. Additionally, the miller’s bran fraction contained a significant amount of peripheral starchy endosperm (sub-aleurone). Using the BFs, this is the first study that was able to investigate the magnitude of the gradient in relative gluten content within the starchy endosperm and how this is affected by cultivar and N-fertilization level. The gradient in relative gluten content, increasing from the inner to outer endosperm (up to 87.3%), was more pronounced without N-fertilization than with N-fertilization. Since 87.3% is an underestimation of the actual relative gluten content in the sub-aleurone, this confirms the hypothesis that the sub-aleurone cells are storage facilities for gluten proteins. Regarding the gradient in gluten composition, without N-fertilization, higher ω- and γ-gliadin proportions and lower B/C-LMW-GS and HMW-GS proportions were observed in the outer than inner endosperm. With N-fertilization, no consistent trends were observed over the different cultivars. This could, at least partly, explain why there is no consensus on the gradient in gluten composition in the literature. Knowledge of the reflection of the protein gradient in the mill streams can be highly valuable for the milling industry. It will aid in comprehending and managing fluctuations in the functionality of specific flour streams, producing specialized flours, coping with low-quality raw materials, and upcycling side streams. This study, along with research on enhancing wheat’s NUE, contributes to a more efficient utilization of N fertilizer. Finally, further research is necessary to understand how environmental factors influence protein gradients and, hence, the composition in mill streams.

## Figures and Tables

**Table 1:** Wheat milling yields of the cultivars Claire (Cl), Apache (Ap), and Akteur (Akt), grown at 0 or 300 kg N ha^−1^ addition levels. BF: break fraction, RF: reduction fraction. Labeling with different letters points out significant differences (Tukey’s HSD test, *p* ≤ 0.05) between the milling yields of different wheat samples.

Mill Fraction	Yield (%)					
	Cl0	Cl300	Ap0	Ap300	Akt0	Akt300
Flour	66.7 ± 1.7 ^AB^	62.8 ± 1.7 ^B^	68.3 ± 0.2 ^A^	69.5 ± 0.4 ^A^	69.5 ± 0.0 ^A^	69.0 ± 0.4 ^A^
BF_total_	33.1 ± 1.5 ^A^	24.7 ± 0.4 ^B^	34.4 ± 1.1 ^A^	22.2 ± 0.4 ^BC^	34.6 ± 0.1 ^A^	20.3 ± 0.1 ^C^
BF1	22.0 ± 1.7 ^A^	14.4 ± 0.1 ^B^	24.4 ± 0.9 ^A^	14.0 ± 0.4 ^B^	25.1 ± 0.3 ^A^	12.8 ± 0.1 ^B^
BF2	6.7 ± 0.6 ^A^	5.9 ± 0.1 ^AB^	6.0 ± 0.1 ^AB^	5.1 ± 0.1 ^BC^	5.6 ± 0.2 ^ABC^	4.7 ± 0.1 ^C^
BF3	4.5 ± 0.4 ^A^	4.5 ± 0.2 ^A^	4.1 ± 0.1 ^A^	3.1 ± 0.0 ^B^	3.9 ± 0.0 ^A^	2.9 ± 0.2 ^B^
RF_total_	33.5 ± 0.2 ^C^	38.6 ± 2.0 ^B^	33.9 ± 1.2 ^C^	47.3 ± 0.0 ^A^	34.9 ± 0.2 ^BC^	48.6 ± 0.5 ^A^
RF1	26.4 ± 3.1 ^D^	33.0 ± 1.3 ^BC^	29.8 ± 0.8 ^CD^	38.0 ± 1.0 ^AB^	29.3 ± 0.4 ^CD^	39.3 ± 0.3 ^A^
RF2	6.4 ± 2.6 ^A^	5.0 ± 0.7 ^A^	3.6 ± 0.5 ^A^	8.0 ± 0.4 ^A^	4.7 ± 0.6 ^A^	8.0 ± 0.1 ^A^
RF3	0.8 ± 0.3 ^A^	0.5 ± 0.0 ^A^	0.5 ± 0.0 ^A^	1.3 ± 0.6 ^A^	0.9 ± 0.0 ^A^	1.3 ± 0.2 ^A^
Miller’s bran	31.5 ± 2.1 ^AB^	32.3 ± 0.3 ^A^	29.5 ± 0.4 ^ABC^	26.7 ± 1.0 ^C^	28.0 ± 0.2 ^BC^	26.8 ± 0.5 ^C^
Shorts	1.8 ± 0.5 ^B^	4.5 ± 1.4 ^A^	2.2 ± 0.3 ^AB^	3.8 ± 0.6 ^AB^	2.5 ± 0.1 ^AB^	4.2 ± 0.1 ^AB^

**Table 2:** Protein composition of the break fractions (BFs) of the cultivars Claire (Cl), Apache (Ap), and Akteur (Akt), without or with N-fertilization (300 kg N ha^−1^). GLIA/GLU: gliadin-over-glutenin ratio, HMW-GS: high molecular weight glutenin subunits, LMW-GS: low molecular weight glutenin subunits (D-type: sulfur-poor, B- and C-type: sulfur-rich). Different letters indicate significant differences (Tukey’s HSD test, *p* ≤ 0.05) in the relative content of a specific protein type between the break fractions of a specific cultivar grown at one level of N-fertilization.

Sample	Mill Fraction	Relative Gluten Content (%)	Gluten Composition (%)							
			ω-Gliadin	α-Gliadin	γ-Gliadin	D-LMW-GS	B/C-LMW-GS	HMW-GS	GLIA/GLU	LMW-GS/ HMW-GS
	BF1	76.61 ± 0.51 ^C^	5.74 ± 0.05 ^B^	31.47 ± 0.36 ^AB^	30.51 ± 0.82 ^B^	0.87 ± 0.04 ^A^	23.61 ± 0.70 ^A^	7.79 ± 0.23 ^A^	2.10 ± 0.09 ^B^	3.14 ± 0.01 ^B^
Cl0	BF2	78.54 ± 0.39 ^B^	5.79 ± 0.05 ^B^	31.88 ± 0.08 ^A^	31.24 ± 0.19 ^B^	0.86 ± 0.02 ^A^	22.83 ± 0.22 ^A^	7.41 ± 0.08 ^AB^	2.22 ± 0.03 ^B^	3.20 ± 0.01 ^A^
	BF3	81.15 ± 0.16 ^A^	6.09 ± 0.04 ^A^	31.12 ± 0.06 ^B^	33.20 ± 0.42 ^A^	0.85 ± 0.01 ^A^	21.64 ± 0.28 ^B^	7.10 ± 0.12 ^B^	2.38 ± 0.05 ^A^	3.17 ± 0.01 ^AB^
	BF1	83.64 ± 0.87 ^A^	7.46 ± 0.19 ^B^	35.73 ± 0.54 ^A^	28.88 ± 1.23 ^A^	0.84 ± 0.04 ^B^	19.51 ± 1.01 ^AB^	7.56 ± 0.39 ^AB^	2.59 ± 0.19 ^AB^	2.69 ± 0.01 ^A^
Cl300	BF2	85.53 ± 0.52 ^A^	7.81 ± 0.07 ^A^	36.16 ± 0.20 ^A^	30.53 ± 0.95 ^A^	0.82 ± 0.06 ^B^	17.80 ± 0.74 ^B^	6.88 ± 0.28 ^B^	2.93 ± 0.16 ^A^	2.71 ± 0.01 ^A^
	BF3	84.37 ± 1.12 ^A^	7.77 ± 0.11 ^AB^	34.03 ± 0.08 ^B^	28.13 ± 1.50 ^A^	1.01 ± 0.04 ^A^	21.15 ± 0.96 ^A^	7.90 ± 0.45 ^A^	2.33 ± 0.16 ^B^	2.81 ± 0.09 ^A^
	BF1	77.45 ± 0.37 ^B^	7.45 ± 0.01 ^B^	33.66 ± 0.32 ^B^	25.14 ± 0.60 ^B^	0.91 ± 0.03 ^A^	25.03 ± 0.66 ^A^	7.82 ± 0.20 ^B^	1.96 ± 0.08 ^B^	3.32 ± 0.01 ^A^
Ap0	BF2	81.20 ± 0.60 ^A^	7.63 ± 0.10 ^A^	34.99 ± 0.44 ^A^	27.22 ± 0.13 ^A^	0.82 ± 0.03 ^A^	22.22 ± 0.46 ^B^	7.13 ± 0.19 ^A^	2.32 ± 0.07 ^A^	3.23 ± 0.02 ^B^
	BF3	82.69 ± 0.70 ^A^	7.59 ± 0.04 ^A^	33.49 ± 0.44 ^B^	28.91 ± 0.84 ^A^	0.84 ± 0.04 ^A^	21.96 ± 0.96 ^B^	7.21 ± 0.31 ^A^	2.34 ± 0.14 ^A^	3.16 ± 0.01 ^C^
	BF1	81.05 ± 0.34 ^C^	8.01 ± 0.16 ^A^	29.85 ± 0.46 ^A^	26.64 ± 0.46 ^A^	0.94 ± 0.02 ^B^	25.64 ± 0.72 ^B^	8.92 ± 0.35 ^B^	1.82 ± 0.08 ^A^	2.98 ± 0.04 ^A^
Ap300	BF2	83.11 ± 0.33 ^B^	8.01 ± 0.18 ^A^	28.01 ± 0.73 ^B^	25.09 ± 0.50 ^B^	1.19 ± 0.08 ^A^	27.53 ± 1.02 ^A^	10.17 ± 0.30 ^A^	1.57 ± 0.09 ^B^	2.82 ± 0.03 ^B^
	BF3	84.46 ± 0.13 ^A^	7.79 ± 0.04 ^A^	26.29 ± 0.20 ^C^	24.74 ± 0.24 ^B^	1.32 ± 0.07 ^A^	29.19 ± 0.34 ^A^	10.67 ± 0.04 ^A^	1.43 ± 0.02 ^B^	2.86 ± 0.04 ^B^
	BF1	78.50 ± 0.21 ^B^	6.10 ± 0.05 ^A^	27.88 ± 0.41 ^A^	21.30 ± 0.76 ^A^	1.20 ± 0.03 ^A^	32.51 ± 0.93 ^A^	11.02 ± 0.24 ^A^	1.24 ± 0.06 ^A^	3.06 ± 0.02 ^A^
Akt0	BF2	80.90 ± 1.00 ^A^	6.52 ± 0.26 ^A^	29.73 ± 1.34 ^A^	23.11 ± 1.41 ^A^	1.08 ± 0.08 ^A^	29.36 ± 2.20 ^A^	10.20 ± 0.72 ^A^	1.47 ± 0.18 ^A^	2.98 ± 0.02 ^B^
	BF3	82.19 ± 0.29 ^A^	6.44 ± 0.02 ^A^	28.46 ± 1.04 ^A^	23.37 ± 1.84 ^A^	1.06 ± 0.07 ^A^	29.96 ± 1.99 ^A^	10.72 ± 0.84 ^A^	1.40 ± 0.17 ^A^	2.90 ± 0.04 ^C^
	BF1	84.50 ± 0.35 ^C^	10.09 ± 0.35 ^A^	28.76 ± 0.86 ^A^	21.54 ± 0.89 ^B^	1.35 ± 0.05 ^A^	26.41 ± 1.38 ^A^	11.85 ± 0.66 ^A^	1.53 ± 0.13 ^A^	2.34 ± 0.02 ^B^
Akt300	BF2	85.78 ± 0.34 ^B^	10.22 ± 0.29 ^A^	28.44 ± 0.70 ^A^	21.92 ± 0.55 ^AB^	1.33 ± 0.05 ^A^	26.70 ± 1.05 ^A^	11.38 ± 0.44 ^A^	1.54 ± 0.10 ^A^	2.46 ± 0.03 ^A^
	BF3	87.29 ± 0.18 ^A^	10.17 ± 0.25 ^A^	28.26 ± 0.51 ^A^	23.45 ± 0.22 ^A^	1.36 ± 0.05 ^A^	25.71 ± 0.58 ^A^	11.05 ± 0.35 ^A^	1.62 ± 0.06 ^A^	2.45 ± 0.04 ^A^

## Data Availability

The data used to support the findings of this study can be made available by the corresponding author upon request.

## Supplementary Materials

The following supporting information can be downloaded at https://www.mdpi.com/article/10.3390/foods12234192/s1, Figure S1: Schematic representation of the Bühler MLU-202 laboratory mill. First, three pairs of corrugated break rolls break up the kernels and scrape off the resulting bran particles to remove the adhering starchy endosperm. Second, three pairs of smooth reduction rollers reduce the particle size of the starchy endosperm particles not ending up in the break fractions. BR: break fraction, RF: reduction fraction.; Figure S2: Chromatograms of (A) albumins and globulins, (B) gliadins, and (C) glutenins separated by modified Osborne fractionation from the first break fraction of Akteur grown at 300 kg N ha^−1^ and analyzed using RP-HPLC. HMW-GS: high molecular weight glutenin subunits, LMW-GS: low molecular weight glutenin subunits.; Figure S3: Wheat kernel protein content on dry base of the cultivars Claire (Cl), Apache (Ap), and Akteur (Akt), grown at 0 or 300 kg N ha^−1^. Different letters indicate significant differences.; Figure S4: Visual representations of models estimating (A) the relative gluten content, (B) GLIA/GLU ratio, and (C) the LMW-GS/HMW-GS ratio in function of the level of N-fertilization, the wheat cultivar and mill fraction. Abbreviations: Cl: Claire, Ap: Apache, Akt: Akteur, GLIA: gliadin, GLU: glutenin, HMW-GS: high molecular weight glutenin subunits, LMW-GS: low molecular weight glutenin subunits, BF: break fraction.
