# Supplementary material for: The Wheat Starchy Endosperm Protein Gradient as a Function of Cultivar and N-fertilization Is Reflected in Mill Stream Protein Content and Composition
Source: Foods. 2023 Nov 21;12(23):4192. doi: 10.3390/foods12234192 (PMC10706271; doi:10.3390/foods12234192)
Supplement: Supplementary file 1 [file foods-12-04192-s001.zip › foods-2689116-supplementary.pdf]

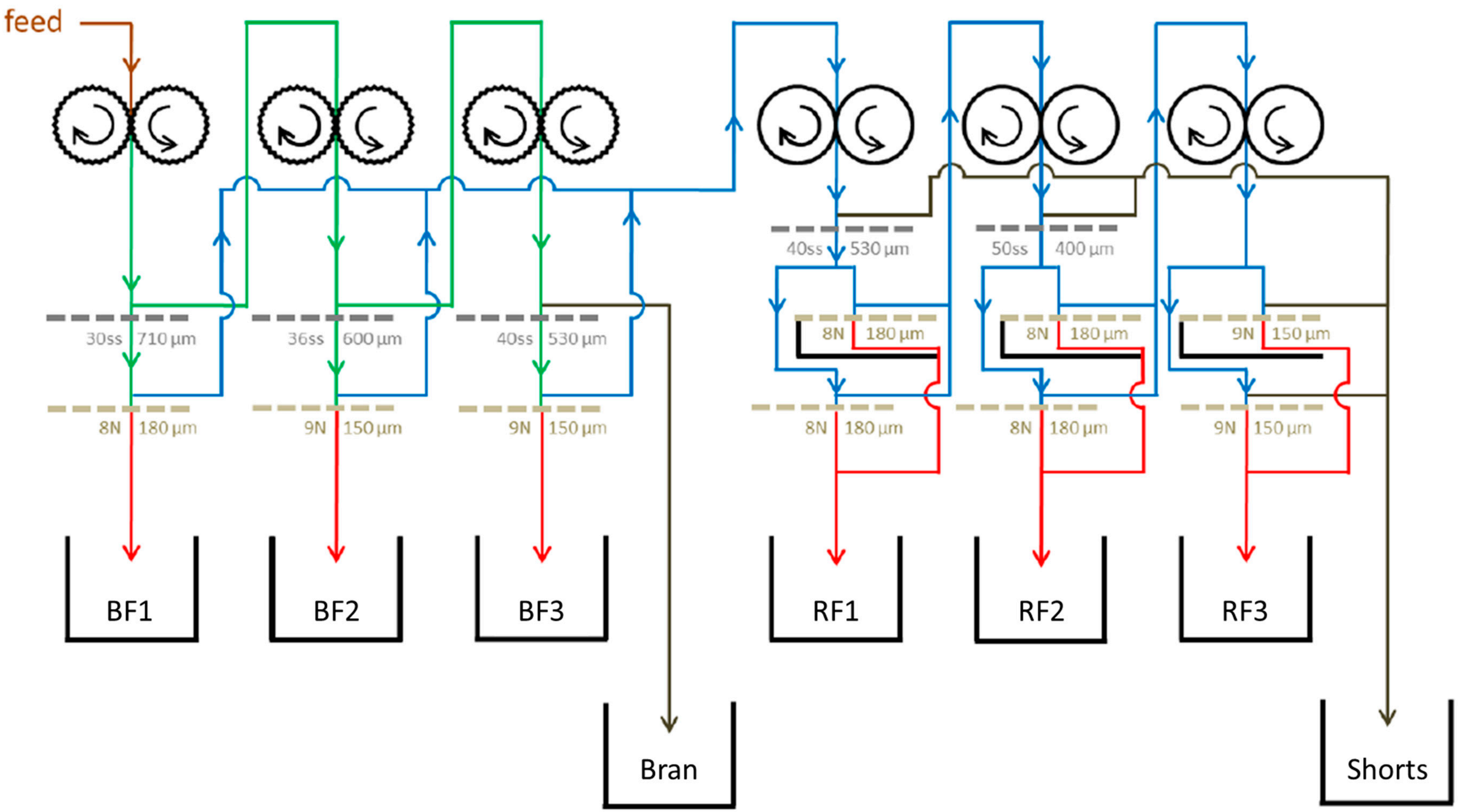

Figure S1: Schematic representation of the Bühler MLU-202 laboratory mill. First, three pairs of corrugated break rolls break up the kernels and scrape off the resulting bran particles to remove the adhering starchy endosperm. Second, three pairs of smooth reduction rollers reduce the particle size of the starchy endosperm particles not ending up in the break fractions. BR: break fraction, RF: reduction fraction.

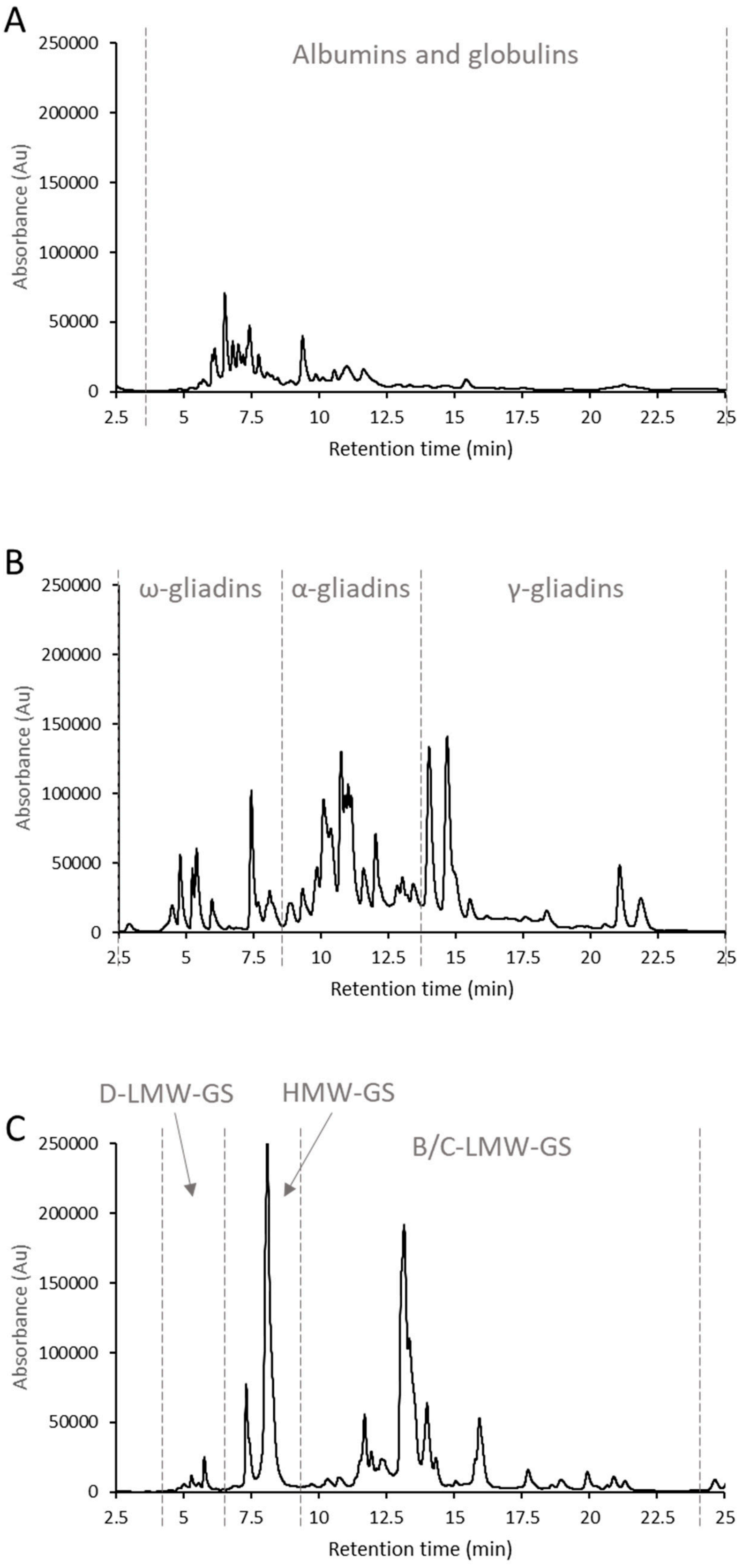
A
250000
200000
150000
100000
50000
0
Absorbance (Au)
Albumins and globulins
2.5
5
7.5
10
12.5
15
17.5
20
22.5
25
Retention time (min)
B
ω-gliadins
α-gliadins
γ-gliadins
C
D-LMW-GS
HMW-GS
B/C-LMW-GS

Figure S2: Chromatograms of A) albumins and globulins, B) gliadins and C) glutenins separated by modified Osborne fractionation from the first break fraction of Akteur grown at 300 kg N ha^{-1} and analyzed using RP-HPLC. HMW-GS: high molecular weight glutenin subunits, LMW-GS: low molecular weight glutenin subunits.

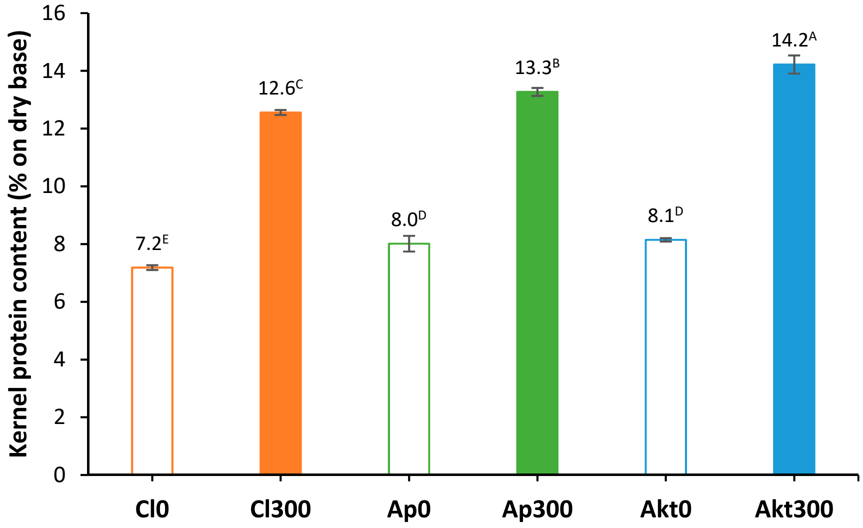

Figure S3: Wheat kernel protein content on dry base of the cultivars Claire (Cl), Apache (Ap) and Akteur (Akt), grown at 0 or 300 kg N ha^{-1}. Different letters indicate significant differences.

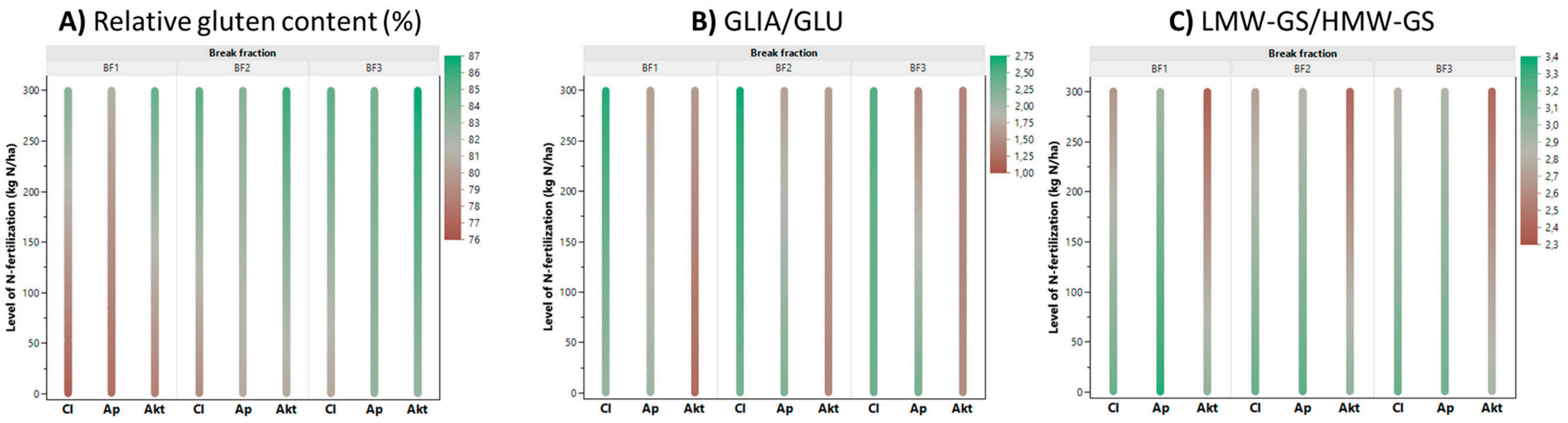

Parameter estimates	Estimates	Prob>|t|
Intercept	79.9211	<0.0001
Break fraction[BF1]	-1.8558	<0.0001
Cultivar[Akt]	1.0743	<0.0001
Cultivar[Ap]	-0.5703	0.0001
Break fraction[BF1]*Cultivar[Ap]	-0.5080	0.0103
Break fraction[BF2]	0.3209	0.0225
Break fraction[BF2]*Cultivar[Akt]	-0.2069	0.2824
Break fraction[BF1]*Cultivar[Akt]	0.1479	0.4367
Break fraction[BF2]*Cultivar[Ap]	0.1287	0.5125
Level of N-fertilization	0.0148	<0.0001
Cultivar[Ap]*Level of N-fertilization	-0.0067	<0.0001
Break fraction[BF1]*Level of N-fertilization	0.0035	0.0003
Cultivar[Akt]*Level of N-fertilization	0.0026	0.0073
Break fraction[BF2]*Level of N-fertilization	0.0005	0.5869
$R^2_{adj} = 0.95$		

Parameter estimates	Estimates	Prob>|t|
Intercept	1.9368	<0.0001
Cultivar[Akt]	-0.4571	<0.0001
Brak fraction[BF2]	0.0712	0.0280
Break fraction[BF1]	-0.0558	0.0770
Cultivar[Ap]	-0.0413	0.1940
Cultivar[Ap]*Level of N-fertilization	-0.0019	<0.0001
Break fraction[BF1]*Level of N-fertilization	0.0007	0.0009
Cultivar[Akt]*Level of N-fertilization	0.0006	0.0037
Break fraction[BF2]*Level of N-fertilization	9.0866e-5	0.6659
Level of N-fertilization	-2.679e-5	0.8560
$R^2_{adj} = 0.89$		

Parameter estimates	Estimates	Prob>|t|
Intercept	3.1282	<0.0001
Cultivar[Akt]	-0.2064	<0.0001
Cultivar[Ap]	0.1582	<0.0001
Break fraction[BF1]*Cultivar[Ap]	0.0706	<0.0001
Break fraction[BF2]*Cultivar[Ap]	-0.0326	0.0109
Break fraction[BF2]*Cultivar[Akt]	0.0277	0.0248
Break fraction[BF1]*Cultivar[Akt]	-0.0179	0.1366
Break fraction[BF1]	0.0166	0.0516
Break fraction[BF2]	-0.0056	0.5122
Level of N-fertilization	-0.0015	<0.0001
Cultivar[Akt]*Level of N-fertilization	-0.0004	<0.0001
Cultivar[Ap]*Level of N-fertilization	0.0003	<0.0001
Break fraction[BF1]*Level of N-fertilization	-0.0002	0.0021
Break fraction[BF2]*Level of N-fertilization	-7.371e-5	0.1980
$R^2_{adj} = 0.98$		

Figure S4: Visual representations of models estimating A) the relative gluten content, B) GLIA/GLU ratio and C) the LMW-GS/HMW-GS ratio in function of the level of N-fertilization, the wheat cultivar and mill fraction. Abbreviations: Cl: Claire, Ap: Apache, Akt: Akteur, GLIA: gliadin, GLU: glutenin, HMW-GS: high molecular weight glutenin subunits, LMW-GS: low molecular weight glutenin subunits, BF: break fraction.
